# FUT3 facilitates glucose metabolism of lung adenocarcinoma via activation of NF-κB pathway

**DOI:** 10.1186/s12890-023-02688-x

**Published:** 2023-11-09

**Authors:** Lanlan Lin, Xiaohui Chen, Guofu Lin, Luyang Chen, Yuan Xu, Yiming Zeng

**Affiliations:** 1 Department of Pulmonary and Critical Care Medicine, The Second Affiliated Hospital of Fujian Medical University, Quanzhou, Fujian Province 362000 China; 2 Fujian Provincial Clinical Research Center of Interventional Respirology, Quanzhou, Fujian Province 362000 China; 3 Fujian Provincial Key Laboratory of Lung Stem Cells, Quanzhou, Fujian Province 362000 China; 4 Clinical Research Center, Quanzhou, Fujian Province 362000 China; 5 School of Public Health, Fujian Medical University, Fuzhou, Fujian Province 350000 China

**Keywords:** FUT3, Lung adenocarcinoma, Glucose metabolism, NF-κB pathway

## Abstract

**Objective:**

Fucosyltransferases (FUTs) molecules have been identified to be involved in carcinogenesis of malignant tumors. Nevertheless, the biological function of fucosyltransferases-3 (FUT3) in lung adenocarcinoma (LUAD) malignant phenotype remains unclear. Herein, we investigated the association between FUT3 and LUAD pathological process.

**Methods:**

Immunochemistry, RT-qPCR and western blot assays were conducted to evaluate the expression of FUT3 in LUAD and corresponding adjacent tissues. The prognostic value of FUT3 was assessed via Kaplan‑Meier plotter database. The biological process and potential mechanism of FUT3 in LUAD were conducted via GSEA. Additionally, immunofluorescence and metabolite activity detection were performed to determine the potential role of FUT3 in LUAD glucose metabolism. The active biomarkers associated with NF-κB signaling pathway were detected via western blot. Subcutaneous tumor model was conducted to analyze the effect of FUT3 on tumorigenesis of LUAD.

**Results:**

FUT3 was remarkably upregulated in LUAD tissues compared with adjacent tissues from individuals. FUT3 overexpression may predict poor prognosis of LUAD patients. Knockdown of FUT3 significantly inhibited tumor proliferation, migration and glucometabolic alteration in LUAD cells. Moreover, GSEA demonstrated that elevated FUT3 was positively related to NF-κB signaling pathway. Additionally, in vitro and in vivo assays also indicated that downregulation of FUT3 resulted in the suppression of oncogenesis and glucose metabolism via inactivation of NF-κB pathway.

**Conclusion:**

Our findings demonstrated that FUT3 was involved in glucometabolic process and tumorigenesis of LUAD via NF-κB signaling pathway. FUT3 may be an optimal target for diagnosis and treatment of LUAD patients.

**Supplementary Information:**

The online version contains supplementary material available at 10.1186/s12890-023-02688-x.

## Introduction

Lung cancer is the prevalent malignancy worldwide [1]. GLOBOCAN data in 2020 demonstrated that lung cancer was the leading cancer mortality (18.0/100,000) and was responsible for secondary cancer incidence (22.4/100,000) annually [2]. Lung adenocarcinoma (LUAD) is the most prevalent histological subtype of non-small cell lung cancer (NSCLC), accounting for approximately 40% of NSCLC cases [3]. The application of molecular targeted therapy and immunotherapy have profoundly improved antineoplastic therapy in lung cancer [4, 5]. While due to the high heterogeneity and mutability of tumors, acquired drug resistance and post-treatment progression are still major clinical challenges [6, 7]. Therefore, it is of great scientific significance to screen sensitive and specific biological markers, and explore the essential mechanism of the occurrence of lung cancer.

Fucosyltransferase III (FUT3), a member of the fucosyltransferase family gene, is located on chromosome 19p13.3. FUT3 encodes an enzyme with both α-(1,3) and α-(1,4) fucosyltransferase activities, and plays a crucial role in cell surface glycosylation [8]. FUT3 was implicated as an essential element in abnormal glucose metabolism. Emerging evidence has indicated that metabolic reprogramming was one of the essential characteristics of tumor cells [9, 10]. Glycolysis not only ensures the energy supply necessary for tumor cells proliferation, but also provides the essential precursors of biomacromolecules and coenzymes for the synthetic metabolism [11, 12]. It has been revealed that FUT3 overexpression enhanced tumorigenesis and malignant potential acquisition. However, the correlation of FUT3 expression with glucose metabolism of lung cancer cells is unclear and remains to be elucidated.

Herein, we investigated the genetic expression and biological function of FUT3 in LUAD tissues. The results indicated that FUT3 was significantly upregulated in cancer tissues and involved in the NF-κB signaling pathway, suggesting that FUT3 dysregulation might play a key role in LUAD pathogenesis and progression. Our study provides a theoretical basis and intervention strategy for the clinical application of FUT3 target medication in lung cancer.

## Materials and methods

### Clinical tissue samples

In total, 32 pairs of human primary lung adenocarcinoma samples and adjacent lung tissues were collected immediately after surgical resection at The Second Affiliated Hospital of Fujian Medical University. All tissue samples were confirmed by histological and pathological examination. None of the patients had received any preoperative chemotherapy or radiotherapy. This study was approved by the Ethics Commission (approval No. 2023 − 111) and all participants signed the written informed consent.

### Immunohistochemistry

Primary tumors were fixed with 4% paraformaldehyde and embedded in paraffin. The deparaffinized and rehydrated tissue specimens were subjected to antigen retrieval in pH 6.0 citrate buffer for 10 min. Then, the endogenous peroxide activity was blocked with 3% hydrogen peroxide. The sections were incubated with the FUT3 primary antibodies (Cat.# DF4068, 1:100; Affinity) overnight at 4℃, and the horseradish peroxidase (HRP) -labeled secondary antibody for 30 min at room temperature. After stained with peroxidase substrate DAB, the slices were observed by Nikon microscope. Images were acquired, and the positive areas were analyzed by using Image J.

### Bioinformatic analysis

Pan-cancer differential expression analysis of FUT3 was performed by Sangerbox (http://sangerbox.com/tool). The prognostic values of FUT3 in lung adenocarcinoma, including overall survival (OS) and first progression (FP), was further assessed via Kaplan-Meier plotter (http://kmplot.com/analysis). We applied the UALCAN dataset to analyze the promoter methylation levels of FUT3 in tumor tissues and adjacent tissues. Furthermore, FUT3 interaction molecules were explored and protein-protein interaction (PPI) network was constructed via GeneMANIA (http://www.genemania.org/).

### Cell culture

Human lung carcinoma cells (A549, H1299, H1975, SPCA-1), bronchial epithelial cells and murine Lewis lung carcinoma (LLC) were obtained from Cell Bank of Chinese Academy of Science (Shanghai, China). The cells were cultured in RPMI-1640 medium (Gibco®, Life Technologies, USA) supplemented with 10% fetal bovine serum (FBS, Gibco) and 1% penicillin-streptomycin (Beyotime, Tianjin, China) in a 5% CO_2_ humidified atmosphere at 37 °C.

### Quantitative real-time PCR

Total RNA of cell lines and tissue samples was extracted using Trizol reagent (Invitrogen, Thermo Fisher Scientific, Inc.) and reversely transcribed into cDNA using the PrimeScript™ RT reagent kit (Takara, Japan). Then, RT-qPCR amplification was conducted using the SYBR Green PCR kit in a Q5 Real-Time PCR System (Thermo Fisher Scientific, USA). GAPDH was used as the internal control and the relative mRNA expression levels were analyzed by the 2^−ΔΔCt^ method. The primer sequences were listed as follows: GAPDH forward, 5ʹ-CACCCACTCCTCCACCTTTG-3ʹ and reverse, 5ʹ-CCACCACCCTGTTGCTGTAG-3ʹ; FUT3 forward, 5ʹ-CTGTCCCGCTGTTCAGAGATG-3ʹ and reverse, 5ʹ- AGGCGTGACTTAGGGTTGGA-3ʹ.

### Western blot

Total cells and tissues proteins were isolated using RIPA lysis buffer and the protein concentration was measured using BCA protein assay (Solarbio, China). All samples were separated by SDS-PAGE gel and electrically transferred to the PDVF membrane (Millipore, Germany). After blocking with 5% skim milk solution for 2 h, the membranes were incubated respectively with primary antibody GAPDH (Cat.# 5174, 1:1000, CST), FUT3 (Cat.# DF4068, 1:500; Affinity), NF-κB (Cat.# ab32536, 1:5000, abcam) and p-NF-κB (Cat.# ab76302, 1:1000, abcam) overnight at 4 °C. Subsequently, the membrane was incubated with the secondary antibodies for 1 h at room temperature. Immunoreactive proteins were visualized using Image Quant LAS 4000 (GE Healthcare Life Science, USA) and quantified using ImageJ software.

### Cell transfection

Control siRNA (si-NC), and FUT3 siRNA (si-FUT3) were obtained from Hanheng (Shanghai, China). 3.0 × 10^5^ cells /well were added into six-well plates overnight before transfection. When the confluence reached 40-50%, cells were transfected with siRNA using Lipofectamine 3000 (Invitrogen, USA) according to the manufacturer’s instructions. After 48 h, cells were collected to identify the efficiency by RT-qPCR. The relevant sequences are as below: si-FUT3, sense 5ʹ-GGACAUGGCCUUUCCACAUTT-3ʹ; anti-sense 5ʹ-AUGUGGAAAGGCCAUGUCCTT-3ʹ; si-NC, sense 5ʹ-UUCUCCGAACGUGUCACGUdTdT-3ʹ; anti-sense 5ʹ-ACGUGACACGUUCGGAGAAdTdT-3ʹ.

### Cell immunofluorescence

Cells were seeded in a 24-well plate at a density of 5.0 × 10^3^ cells/well, fixed with 4% paraformaldehyde for 15 min and permeabilized with 0.5% Triton X-100 for 5 min. After blocked with 5% fetal bovine serum for 1 h, cells were incubated with primary antibodies of GLUT1 (Cat.# ab115730, 1:200, abcam) and LDHA (Cat.# ab47010, 1:1000, abcam) overnight at 4 °C. The next day, cells were incubated with the corresponding secondary antibodies for 1 h at room temperature in the dark, and were counterstained with DAPI for 15 min. Immunofluorescence was visualized and photographed under a fluorescence microscope (Nikon, Japan).

### 5-Ethynyl-2’-deoxyuridine (EdU) assay

Cells was adjusted to the density of 2.0 × 10^4^ cells/well and added into 24-well plates. After co-cultured with EdU working solution (1:1000) for 2 h in a humidified incubator at 37 °C, cells were immobilized with methanol for 15 min and permeabilized with 0.5% Triton X-100 for 5 min. Subsequently, cells were incubated with Click reaction solution for 30 min, followed by stained with DAPI for 15 min. The images were captured with Nikon microscope (Nikon, Japan) and cell counting was calculated by ImageJ software.

### Transwell migration assay

Cell migrative ability was evaluated by transwell migration assays. Cells were resuspended in a serum-free medium and seeded at 5.0 × 10^4^ cells/well into 12-well plates. Medium containing 20% FBS was placed into the lower chamber. After 24 h, non-migrated cells on the upper chambers were scraped out with cotton swab. Migrated cells were fixed in 4% paraformaldehyde for 30 min and stained with 1% crystal violet for 30 min. Cells were observed by microscope (Nikon, Japan) in randomly selected fields.

### Glucose 6-phosphate dehydrogenase (G6PDH) activity assay

G6PDH activity was detected using G6PDH activity assay kit (Solarbio, Cat.# BC0260) following the manufacturer’s protocol. Cell lysates were prepared and then a combination of the reagents provided by the kit was applied to prepare a working solution. Subsequently, the optical density (OD) change within 5 min at 340 nm of each sample was measured by spectrophotometer and G6PDH activity was calculated using the absorbance coefficient of NADPH.

### Lactate dehydrogenase (LDH) activity assay

A lactate dehydrogenase activity assay kit (Solarbio, Cat.# BC0680) was used for LDH measurements. After collecting cell lysates, lactate assay buffer, substrate mix and enzyme reagents were mixed and added into cell lysates. Mixture was incubated for 3 min at room temperature, and the OD 450 nm of each sample was measured by spectrophotometer and the concentration of LDH was calculated according to a standard curve and computational formula.

### Subcutaneous tumor model

A subcutaneous tumor model was set up in C57BL/6J mice and all experimental procedures were approved by The Second Affiliated Hospital of Fujian Medical University. C57BL/6J mice (6 weeks old, male, n = 16) were purchased from SLAC Laboratory Animal Co., Ltd (Shanghai, China). 5.0 × 10^6^ LLC cells were subcutaneous implanted into the right dorsal flank of mice and after cell implantation, the body weight and tumor volumes were monitored every three days. Mice rendered tumor-bearing were divided randomly into 4 groups and treated by intratumor injection of si-NC, si-FUT3 or NF-κB activator Asatone every three days. Mice were anesthetized by intraperitoneal injection of sodium pentobarbital before sacrifice. Tumor volume was calculated by formula [length × width (mm)^2^]/2. The mice were euthanized and tumor tissues were photographed.

### KEGG and Gene set enrichment analysis (GSEA)

Kyoto Encyclopedia of Genes and Genomes (KEGG) (https://www.kegg.jp/kegg/kegg1.html) pathway was performed with an enrichment p value < 0.05 [13]. Java GSEA software v4.1.0 (http://www.broadinstitute.org/gsea) was implemented for gene set enrichment analysis and enrichment map analysis was further applied for the results. The *P* value < 0.05 and a false discovery rate (FDR) q value < 0.25 were set as a criterion and regarded as significant enrichment.

### Statistical analysis

Statistical analyses and graphics generation were conducted using GraphPad Prism 7.0. Comparisons of quantitative data were conducted using Student’s t-test or one-way ANOVA. In addition, two-way ANOVA followed by Bonferroni’s multiple mean comparisons were applied to analyze group differences. *P* < 0.05 was considered as statistical significance.

## Results

### The differential expression and prognostic value of FUT3 in LUAD tissues

**Fig. 1:**
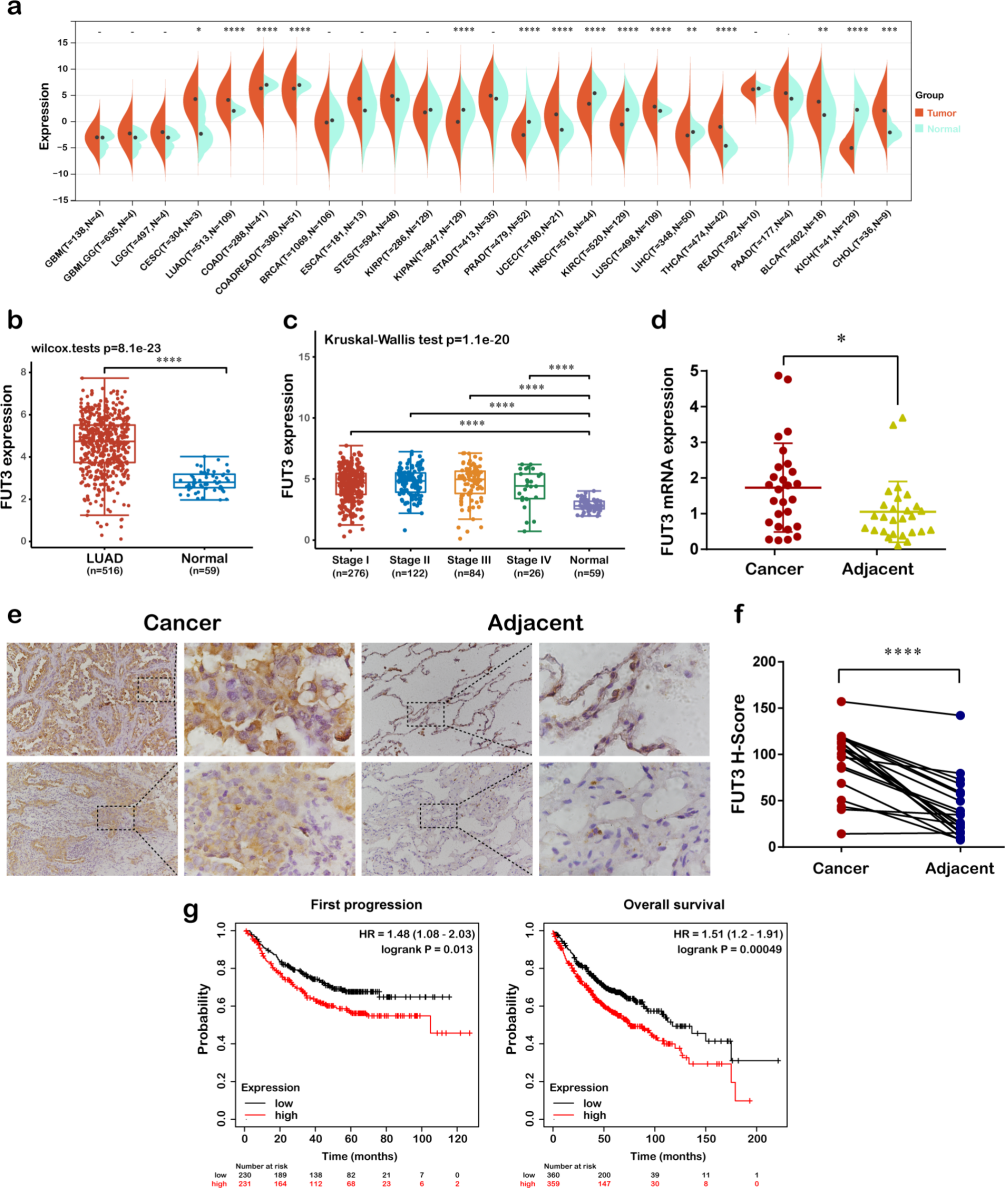
Differential expression and prognostic value of FUT3 in LUAD patients. **(a)** Pan-cancer analysis of FUT3 expression by Sangerbox database. **(b)** The FUT3 mRNA levels in LUAD tissues compared with the normal pulmonary tissues from TCGA. **(c)** Expression of FUT3 in different stages of LUAD by TCGA. **(d)** The FUT3 mRNA expression of LUAD tissues and adjacent tissues was detected via RT-qPCR. **(e)** Immunohistochemistry analysis of FUT3 expression in LUAD tissues and corresponding adjacent tissues. **(f)** FUT3 histological score in tumor tissues and adjacent tissues with immunohistochemistry. **(g)** Kaplan-Meier analysis of FUT3 expression with first progression (FP) and overall survival (OS) in LUAD patients. (*P < 0.05, **P < 0.01, ***P < 0.001, ****P < 0.0001)

We employed Sangerbox database to investigate the expression of FUT3 in pan-cancer. As shown in Fig. 1a, FUT3 was upregulated in the majority of malignancies including LUAD. The TCGA data sets were applied to reveal the expression of FUT3 in LUAD. Compared with normal tissues, the expression level of FUT3 was remarkably elevated in LUAD tissues and exhibited distinct elevation in different stages of LUAD (Fig. 1b-c). To further verify the accuracy of the above results, RT-qPCR and IHC staining experiments were performed. The RT-qPCR data revealed that FUT3 was upregulated in LUAD when compared with adjacent tissues (Fig. 1d). A similar tendency of IHC staining was also observed. The FUT3 H-score in tumor tissues was significantly higher than that in adjacent tissues (Fig. 1e, f).

Subsequently, we analyzed the prognostic values of FUT3 in lung cancer. Figure 1 g indicated that high expression of FUT3 in LUAD predicted worse survival for overall survival (HR = 1.51, logrank *P* < 0.001) and first progression (HR = 1.48, logrank *P* = 0.013). These data suggested that FUT3 expression might be an unfavorable factor for the prognosis in LUAD.

### FUT3 methylation and function enrichment in LUAD

**Fig. 2:**
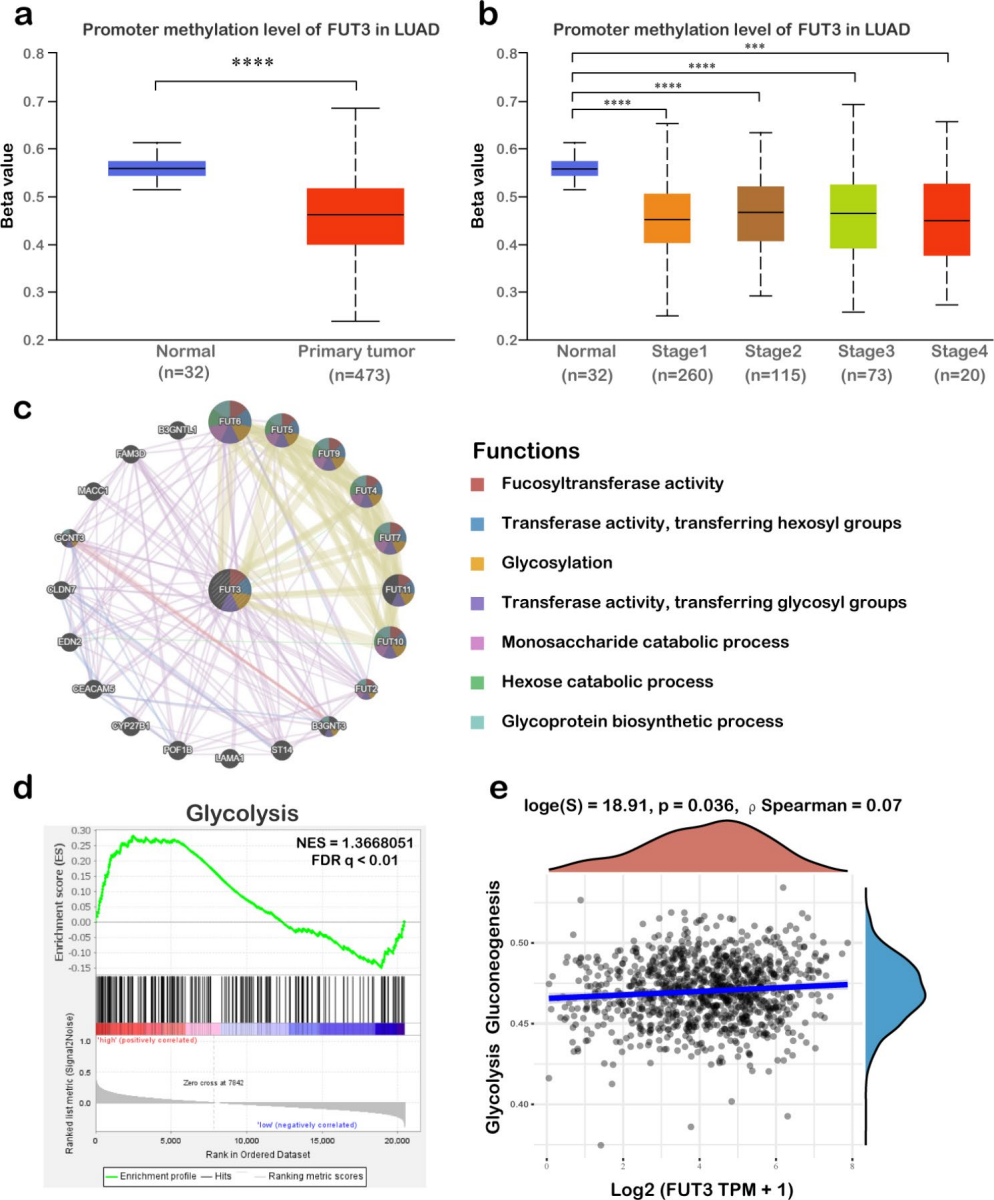
Promoter methylation and function enrichment analysis of FUT3. **(a-b)** Promoter methylation level of FUT3 in primary LUAD tumors and different stages of LUAD from UALCAN database. **(c)** Protein interaction network of FUT3 constructed by GeneMANIA. **(d-e)** Association of FUT3 with glycolysis. (***P < 0.001, ****P < 0.0001)

Epigenetic regulation of gene expression relies on DNA methylation [14]. We evaluated the FUT3 promoter methylation level in 32 healthy lung tissues and 473 primary LUAD tissues. The data revealed that normal tissues exhibited a significant downregulation of FUT3 promoter methylation compared with LUAD tumors (Fig. 2a). Furthermore, FUT3 promoter methylation was reduced in different stage of LUAD (Fig. 2b).

We investigated the possible protein targets and functions of FUT3 in LUAD via GeneMANIA. The network revealed that FUT3 co-expressed mostly with FUT6, FUT5, and FUT9. These proteins were predicted to participate in the fucosyltransferase activity, transferase activity and glycosylation process according to the prediction (Fig. 2c). We further performed GSEA to investigate the potential mechanisms of FUT3-related biological process in LUAD (Fig. 2d). The results showed that FUT3 was positively correlated with glycosylation (NES = 1.37, FDR q < 0.01). In addition, the correlation between FUT3 genetic expression and pathway scores was analyzed by Spearman correlation. The results demonstrated that FUT3 was associated with glycolysis and gluconeogenesis (Fig. 2e).

### FUT3 promoted LUAD migration and proliferation

**Fig. 3:**
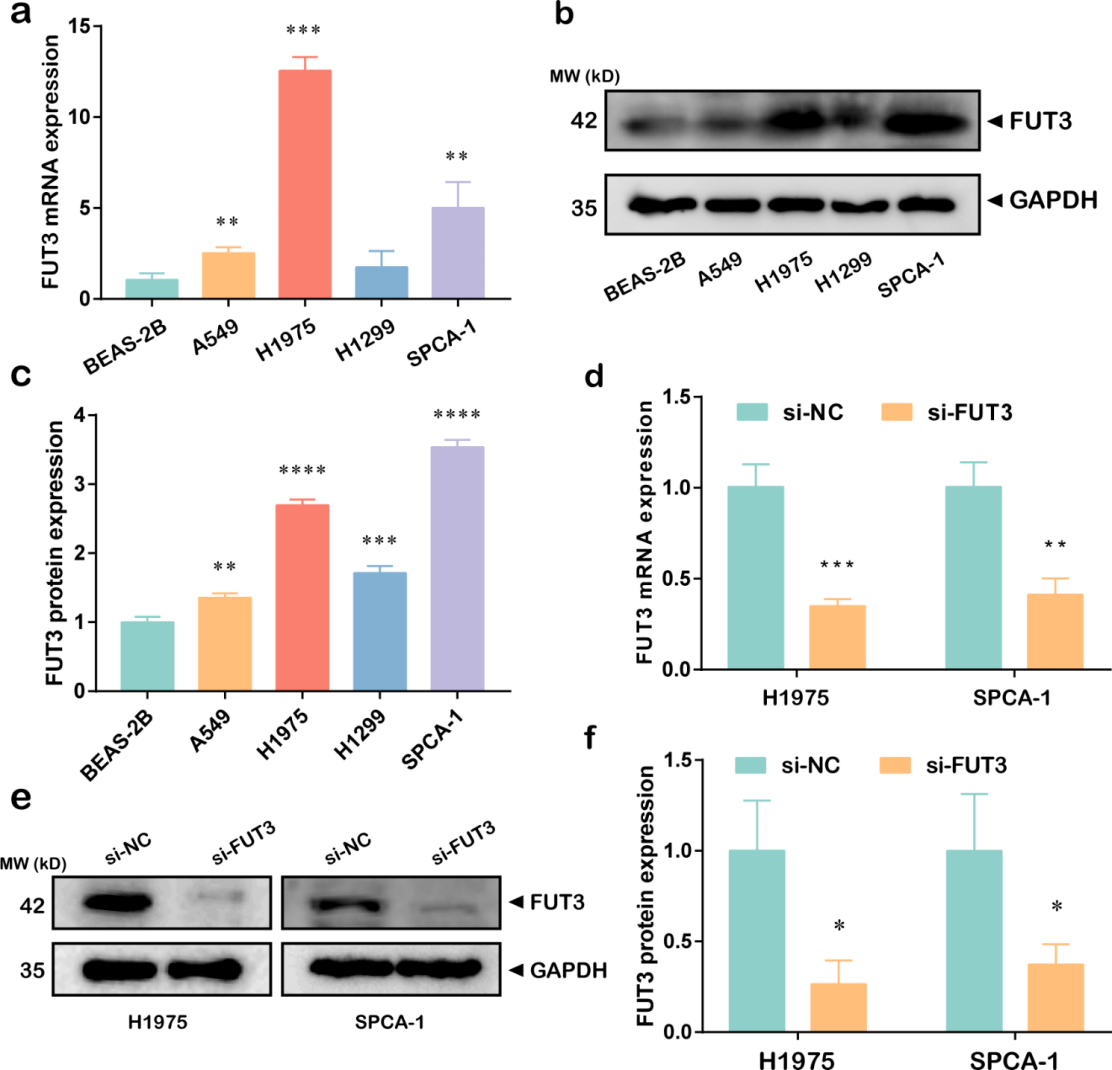
FUT3 expression in LUAD cell lines. **(a-c)** FUT3 mRNA and protein expressions in the normal pulmonary epithelial cell and LUAD cell lines were evaluated by RT-qPCR and western blot. **(d-f)** RT-qPCR and western blot analysis were performed to detect FUT3 expression after transfection of FUT3 siRNA in H1975 and SPCA-1 cells. (*P < 0.05, **P < 0.01, ***P < 0.001, ****P < 0.0001)

**Fig. 4:**
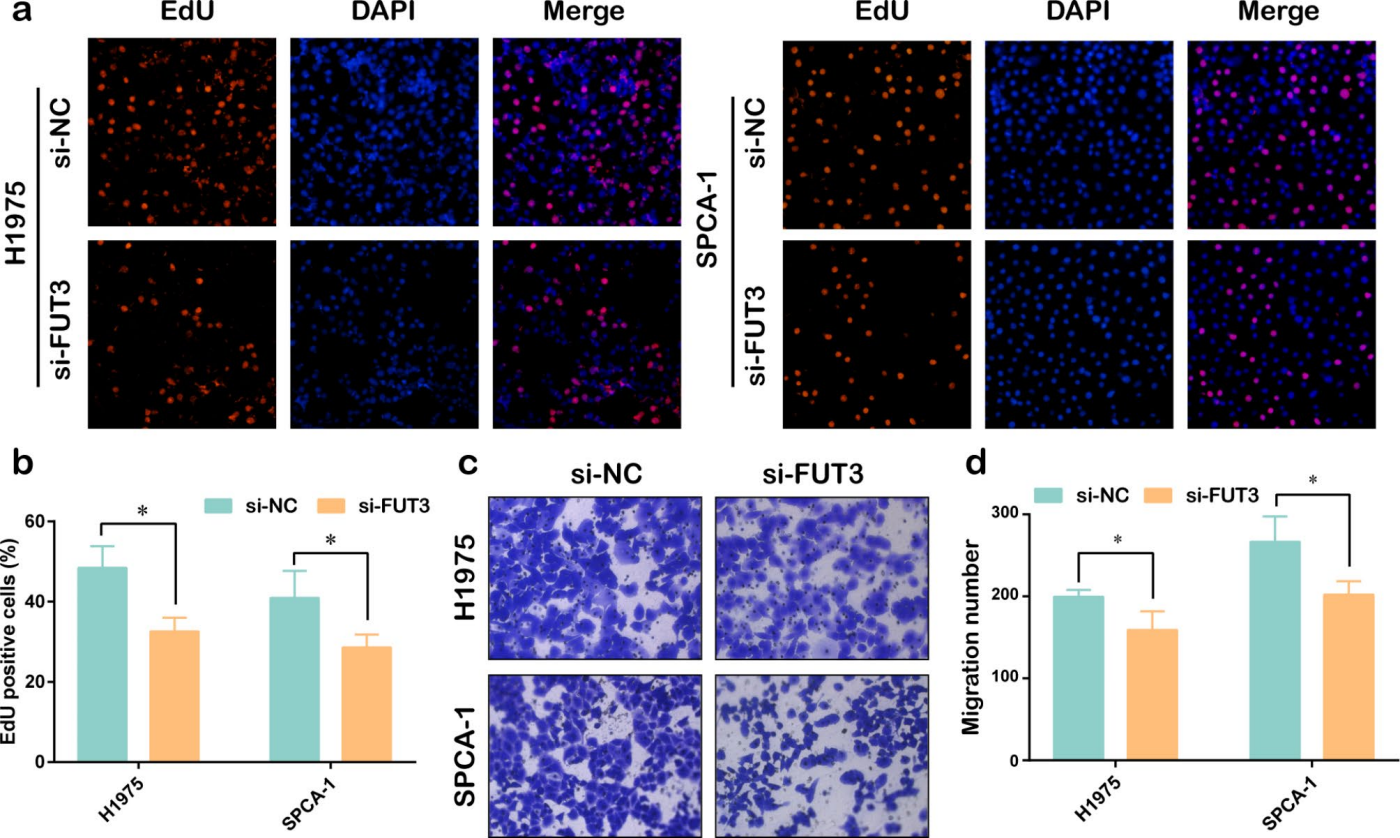
Downregulation of FUT3 inhibited the proliferation and migration of LUAD cell lines. **(a-b)** EdU staining assay was conducted to evaluate the proliferation of FUT3-downregulated LUAD cells, scale bar 50 μm. **(c-d)** Transwell migration assay was performed after transfection with si-FUT3 in H1975 and SPCA-1 cells, scale bar 50 μm. (*P < 0.05)

To investigate the effect of FUT3 on LUAD progression, we examined the expression of FUT3 in LUAD cell lines and normal bronchial epithelial cell line BEAS-2B. The results indicated that FUT3 mRNA and protein expressions were elevated in LUAD cells, especially in H1975 and SPCA-1 cells (Fig. 3a-c). Therefore, FUT3 siRNA was applied to H1975 and SPCA-1 cell lines. RT-qPCR and western blot presented that FUT3 expression was notably downregulated in cells transfected with si-FUT3 (Fig. 3d-f), indicating that the silencing efficacy of si-FUT3 was effective. We further assessed proliferative ability with EdU assay and observed that FUT3 silencing might impair the percentage of EdU-positive cells in LUAD cell lines (Fig. 4a-b). Similarly, the migration was also decreased by downregulation of FUT3 in both H1975 and SPCA-1 cells (Fig. 4c-d).

### FUT3 was involved in LUAD glucose metabolism

**Fig. 5:**
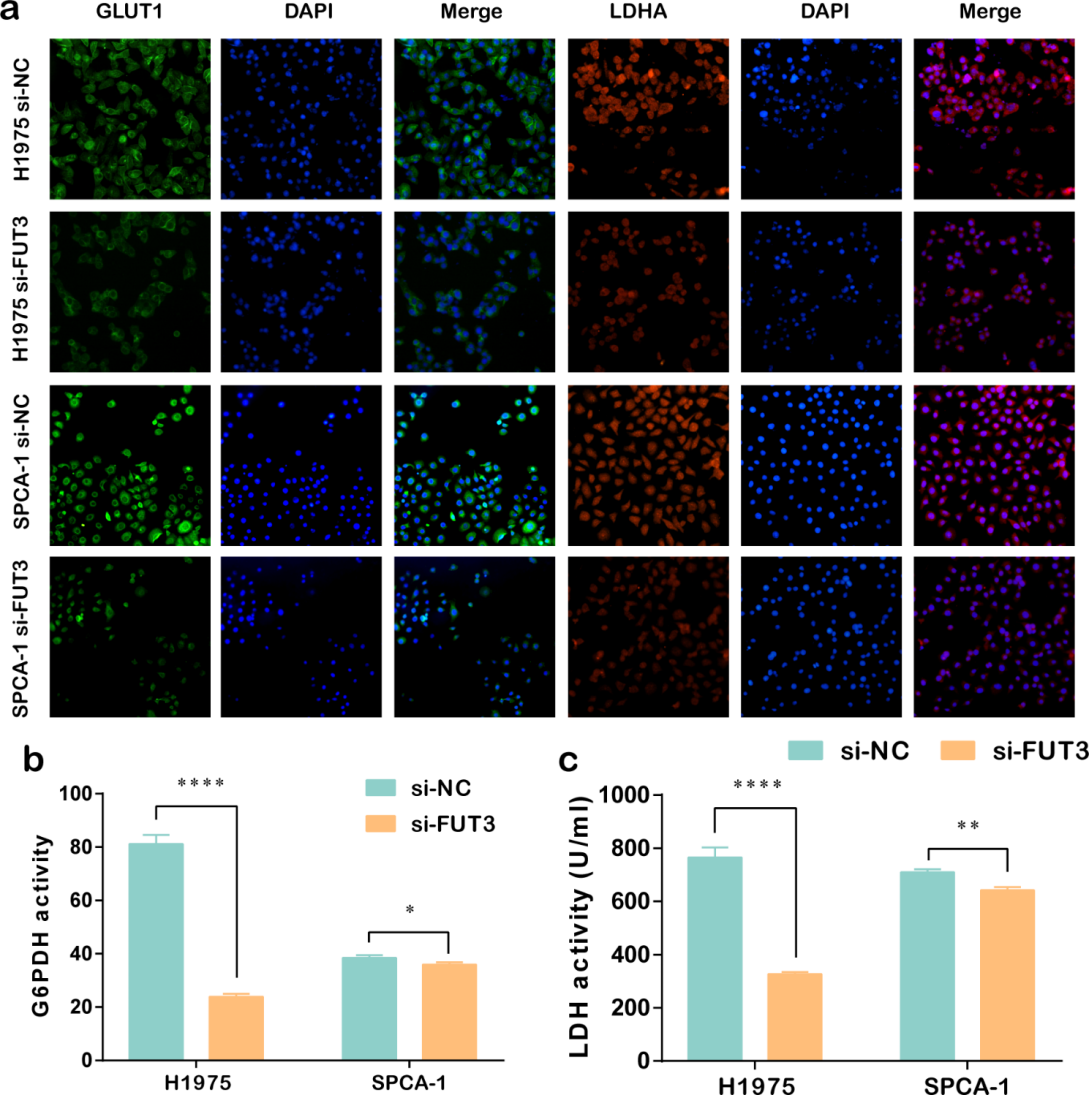
FUT3 participated in glucose metabolism in LUAD cells. **(a)** GLUT1 and LDHA immunofluorescence was performed to present the alteration of glucose metabolism in H1975 and SPCA-1 cells after FUT3 knockdown. **(b-c)** The enzyme activity of G6PDH and LDH was measured in LUAD cells. (*P＜0.05, **P < 0.01, ****P < 0.0001)

Previous studies have reported that metabolic dysfunction was considered as a crucial driving factor for cancer progression, and providing energy and glucose for neoplastic proliferation [15]. Based on the GSEA enrichment and Spearman correlation analysis, we speculated whether glucose metabolism alteration occurred in FUT3-regulated biological function in LUAD cells. We detected essential molecules of glucose metabolism such as glucose transporter 1 (GLUT1) and lactate dehydrogenase A (LDHA). Consequently, immunofluorescence experiments in LUAD cells confirmed that lower level of FUT3 could reduce expression levels of GLUT1 and LDHA (Fig. 5a). Moreover, we conducted glucose uptake and lactate production assays to verify whether FUT3 regulates the glucose metabolism of LUAD cells. Results showed that FUT3 downregulation reduced both glucose uptake and lactate production, which suggested a decline in cell glucose metabolism (Fig. 5b-c).

### FUT3 facilitated glucose metabolism via activation of the NF-κB pathway

**Fig. 6:**
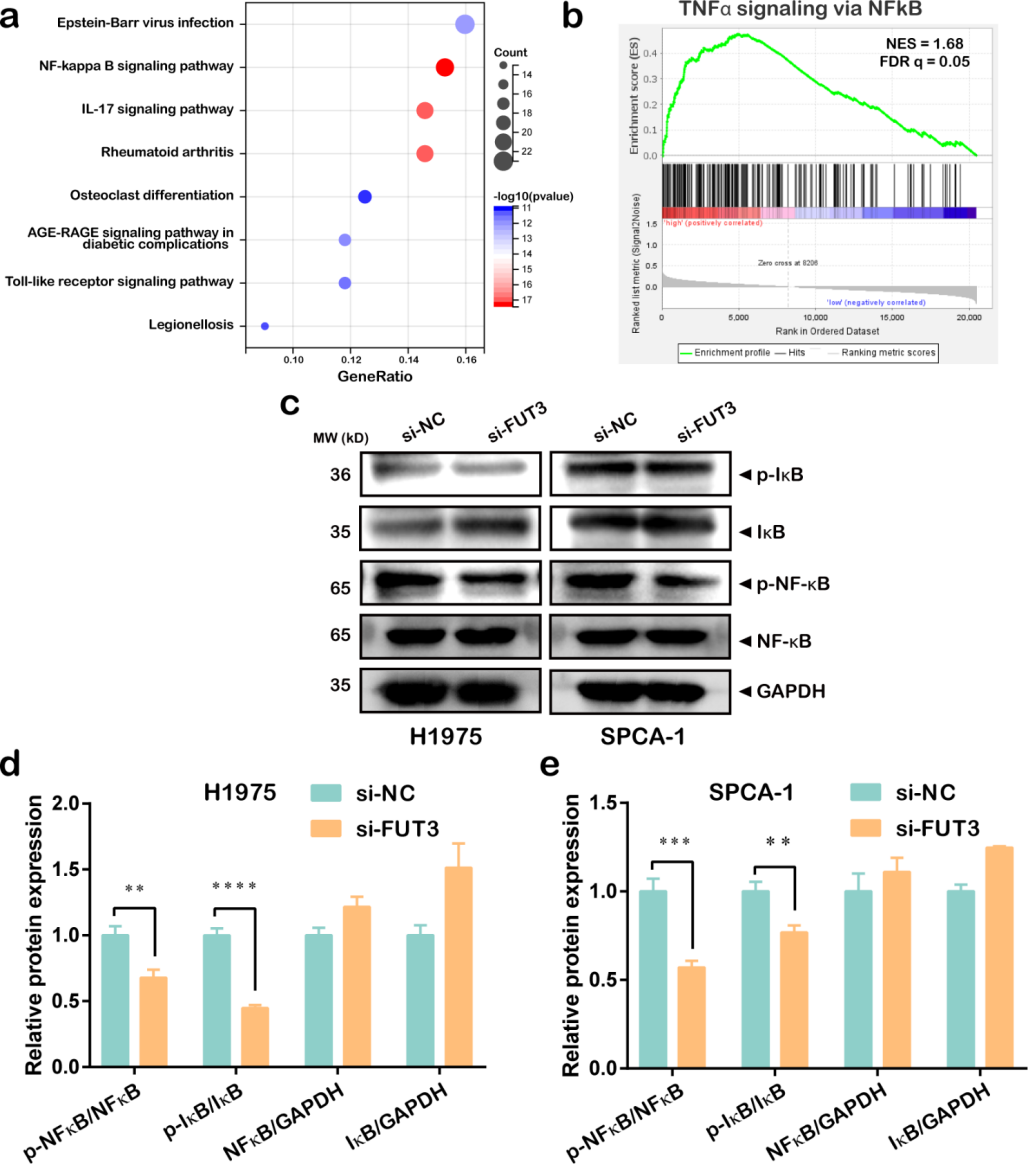
FUT3 was correlated with NF-κB pathway. **(a-b)** FUT3 related differential gene enrichment analysis was performed via KEGG database and GSEA. **(c-e)** Essential molecules’ expressions of NF-κB pathway were measured by western blot after transfection with si-FUT3 in H1975 cells and SPCA-1 cells. (**P < 0.01, ***P < 0.001, ****P < 0.0001)

Differential gene enrichment analysis was performed to excavate signaling pathways that associated with FUT3 expression in the glucose metabolism. The results revealed that FUT3 might be involved in LUAD progression through NF-κB signaling pathway (Fig. 6a). GSEA also indicated FUT3 was positively correlated with TNFα signaling via NF-κB (Fig. 6b). The above evidence suggested that FUT3 might manifest its unique function via the NF-κB pathway. To further investigate whether FUT3 mediated metabolism through the activation of NF-κB pathway, the essential molecules of NF-κB pathway in H1975 and SPCA-1 cells were detected by western blot. The results presented that the expression levels of p-NF-κB and p-IκB were reduced (Fig. 6c-e).

### Inactivation of FUT3 suppressed the growth and glucose metabolism of LUAD in vivo

**Fig. 7:**
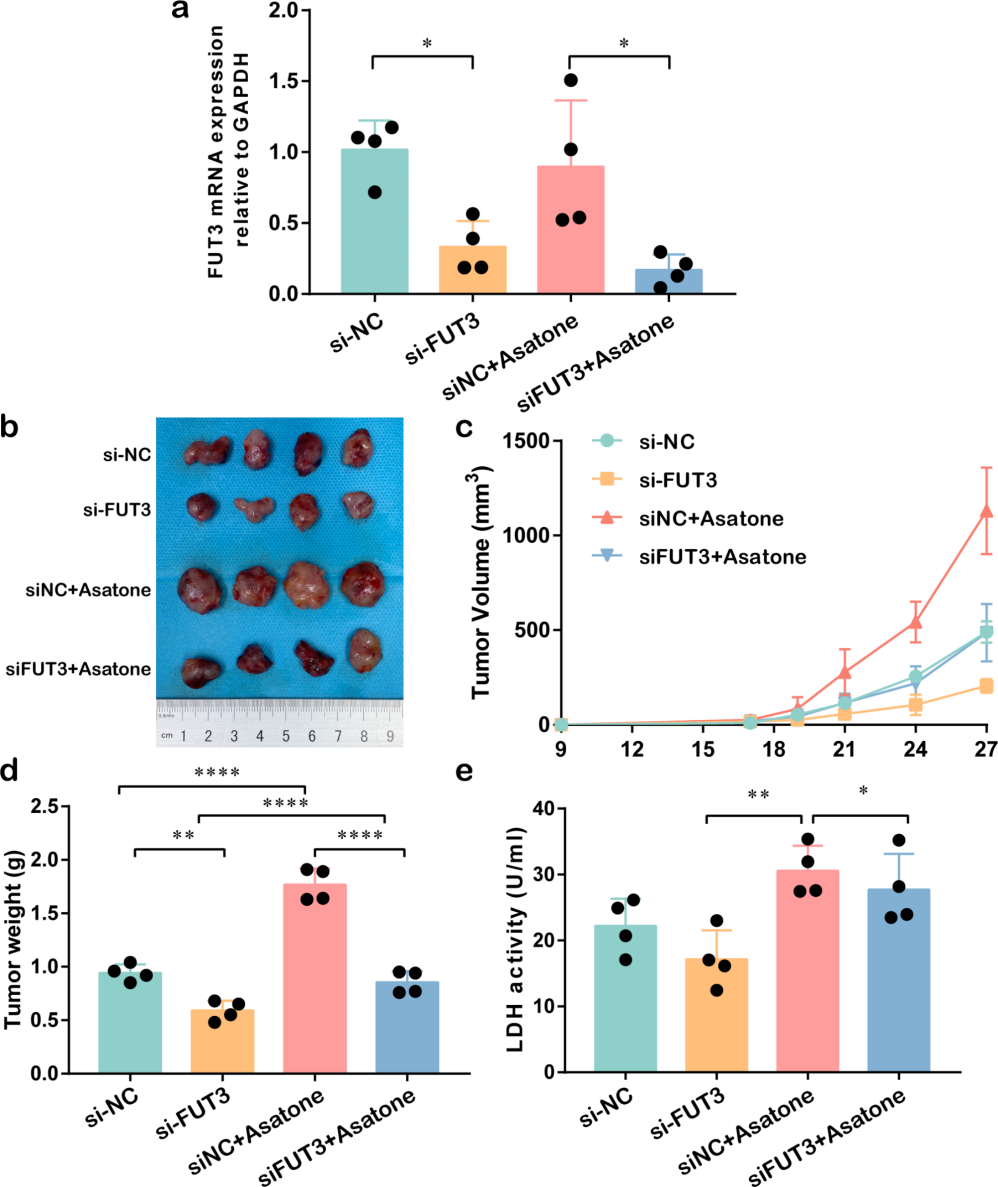
Effect of FUT3 on the tumor growth and glucometabolic alteration of Lewis lung carcinoma cells (LLC) in vivo. **(a)** FUT3 mRNA expression in the C57BL/6J was evaluated by RT-qPCR. **(b)** Representative images of subcutaneous tumors dissected from C57BL/6J mice in different groups. **(c)** The growth curve showed the subcutaneous tumor of C57BL/6J mice injected with LLC cells with or without si-FUT3 and Asatone. **(d)** Comparison of tumor weight from different groups of C57BL/6J mice. **(e)** Glucometabolic changes in tumor tissues was detected by LDH activity assay. (*P < 0.05, **P < 0.01, ****P < 0.0001)

We validated the FUT3 potential tumorigenicity on LUAD in vivo using a mouse subcutaneous tumor model. We subcutaneously injected LLC cells into the right dorsal flank of C57BL/6J mice. After 27 days, the mice were euthanized and tumors were immediately harvested. In our study, si-NC and si-FUT3 were modified with methoxy and cholesterol to ensure the high activity of the siRNAs in vivo. As demonstrated in Fig. 7a, FUT3 mRNA expressions were significantly downregulated in si-NC, and si-NC with Asatone group when compared with control groups. Figure 7b displayed the subcutaneous tumor with si-NC, si-FUT3 or Asatone intratumorally. The result indicated tumors in FUT3-downregulated groups were minor than control groups, while NF-κB activator Asatone might reverse the tumor volume (Fig. 7c). Additionally, the tumor weight presented a similar trend (Fig. 7d). Moreover, LDH activity test was applied to evaluate the tumor glucose metabolism. The result demonstrated that downregulation of FUT3 reduced LDH activity while Asatone restored the change in the resected tumor when compared with the control groups (Fig. 7e).

## Discussion

Abnormal glucose metabolism entails profound changes in the protein expression involved in the proliferation process [16]. Herein, we investigated the potential role of FUT3 in LUAD progression. The consequences indicated that FUT3 was remarkably overexpressed and predicted poor clinical outcome in LUAD. Downregulation of FUT3 inhibited glucose metabolism, as evidenced by the downregulation of G6PDH and LDH activity. Emerging evidence has indicated that FUT3 enhanced migration and tumorigenesis of lung cancer [17], but the biological process of FUT3 in LUAD glucose metabolism was not been previously verified.

The glycoproteins on the membrane of cancer cells have altered oligosaccharide chains when cells evolve into cancerous [18]. The abnormal glycosylation may affect the regular function of cells. It has been reported that glycosyltransferase participated in the synthesis of complex glycochains [19]. Previous studies have reported FUTs molecules were involved in glucose metabolism of cancer cells. FUT8 was elevated in cancers including liver cancer, pancreatic cancer and lung cancer [20–23]. Noda et al. demonstrated that FUT8 and fucosylated alpha-fetoprotein have been applied as diagnostic markers for liver cancer [24]. Additionally, FUT7 might modulate the immune microenvironment of triple negative breast cancer via glycolysis regulation [25], indicating the role of FUTs in regulation of cell conjunction and metastatic process.

Since FUT3 was described as an essential member of FUTs, we supposed whether FUT3 might be involved in proliferation and glucose metabolism in LUAD cells. In the present study, we demonstrated that FUT3 was elevated in LUAD tissues, and positively associated with proliferative abilities and glucose metabolism. Similarly, Hirakawa et al. reported that FUT3 expression was increased in pancreatic cancer and colorectal cancer, downregulation of FUT3 inhibited the fucosylation of TGF-β, and impaired the capacity of epithelial-mesenchymal transition [17, 26]. Elevated FUT3 expression was also observed in oral squamous cell carcinoma, breast infiltrating ductal carcinoma, and ovarian cancer [27, 28]. Previous studies have indicated that FUT3 played an essential role in regulating cell cycle and proliferation-related signaling pathways in tumors [29, 30]. Therefore, downregulation of FUT3 might inhibit the proliferation of lung adenocarcinoma cells by affecting cell cycle and proliferation-related pathways. Moreover, FUT3 may alter the expression of Lewis antigen. Lewis antigen may be further post-translationally modified into glycoproteins CA19-9 [31], and decrease the adhesion of cancer cells [32]. Additionally, He et al. also demonstrated that FUT3 also participated in epithelial-mesenchymal transition process via regulation of cell surface glycosylation [33]. Our study consistently revealed that abnormal FUT3 expression could regulate the glucose metabolism of LUAD cells. However, the specific mechanism remains further verified.

The aberrant activation of NF-κB pathway may modulate many cellular processes including autophagy, apoptosis, metastasis and energy metabolism [34]. In this study, we identified a link between the NF-κB pathway and FUT3. The result identified that downregulation of FUT3 inhibited LUAD glucose metabolism by modulating the NF-κB pathway. Various studies have indicated that the NF-κB signaling pathway may modulate intracellular glucose metabolism through diverse mechanisms, including the regulation of glucose transport, glycolytic pathways, and glucose synthesis [35–37]. Emerging evidence has indicated that FUTs genes may participate in the metabolism of tumor cells by regulating the activity of the NF-κB signaling pathway. For instance, FUT4 regulated epithelial mesenchymal transition of breast cancer cells via activation of PI3K/Akt and NF-κB signaling pathways [38]. Moreover, FUT4 significantly modulated NF-κB pathway and affected the progression of osteoarthritis [39]. Lin et al. also reported that FUT8 might further affect osteosarcoma by remodeling TNF/ NF-κB signaling [40], The above evidence was consistent with our results that FUT3 up-regulated NF-κB activity in LUAD cells.

The aim of the present study was to evaluate the biological effect and potential mechanism of FUT3 as an initial exploration. However, there were some limitations in our study. Firstly, there was a limited quantity of clinical samples and prognosis information of LUAD patients was not collected, which is partly due to the access of specimen and data. Secondly, the direct involvement of FUT3 in glucose metabolism remains undetected and the underlying mechanism of FUT3 regulating the process of LUAD remains unexplored. In addition, immunochemistry experimental of subcutaneous tumor models and FUT3 knockout mice models were not conducted, which need to be further identified.

## Conclusion

In summary, our current study demonstrated that FUT3 was remarkably upregulated in LUAD tissues and predicted poor clinical prognosis. Downregulation of FUT3 could inhibit the proliferation and glucose metabolism via NF-κB pathway. Our results indicated antagonist of FUT3 might be an optional remedy for LUAD patients than traditional opioids.

### Electronic supplementary material

Below is the link to the electronic supplementary material.

Supplementary Material 1

## Data Availability

All data generated or analyzed in present study are included in this published article.

## Abbreviations

EdU: 5-Ethynyl-2’-deoxyuridine
FBS: Fetal bovine serum
FP: First progression
FUT3: Fucosyltransferase III
G6PDH: Glucose 6-phosphate dehydrogenase
GLUT1: Glucose transporter 1
GSEA: Gene set enrichment analysis
HRP: Horseradish peroxidase
LDH: Lactate dehydrogenase
LDHA: Lactate dehydrogenase A
LLC: Lewis lung carcinoma
LUAD: Lung adenocarcinoma
NSCLC: Non-small cell lung cancer
OS: Overall survival
PPI: Protein-protein interaction
