# Supplementary material for: FUT3 facilitates glucose metabolism of lung adenocarcinoma via activation of NF-κB pathway
Source: BMC Pulm Med. 2023 Nov 9;23:436. doi: 10.1186/s12890-023-02688-x (PMC10636925; doi:10.1186/s12890-023-02688-x)

Original Western blot Figure

Lanlan Lin[1,2#], Xiaohui Chen[1,2#], Guofu Lin[1,2#], Luyang Chen[1,2], Yuan Xu[1,2,3 *], Yiming Zeng[1,2,3 *]

1. Department of Pulmonary and Critical Care Medicine, The Second Affiliated Hospital of Fujian Medical University, Quanzhou, Fujian province, 362000, China

2. Respiratory Medicine Center of Fujian Province, Quanzhou, Fujian province, 362000, China

3. Clinical Research Unit, The Second Affiliated Hospital of Fujian Medical University, Quanzhou, People's Republic of China

Figure 3b

GAPDH BEAS-2B--A549--H1975--H1299--SPCA-1

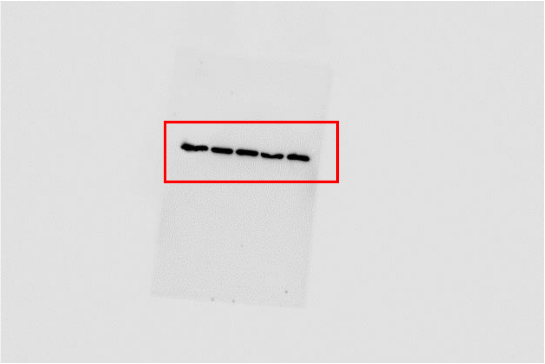

FUT3 BEAS-2B--A549--H1975--H1299--SPCA-1

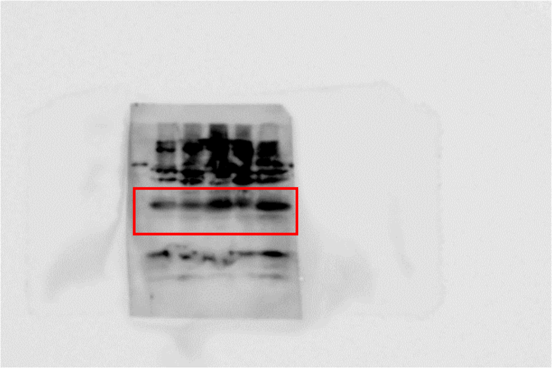

Figure 3e

GAPDH H1975 siNC/siFUT3--SPCA-1 siNC/siFUT3

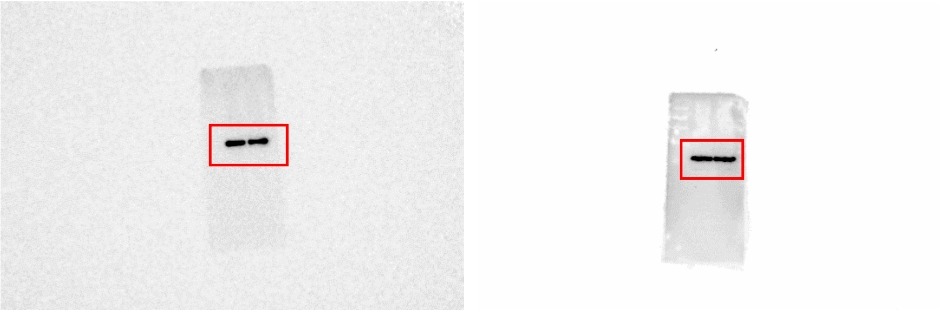

FUT3 H1975 siNC/siFUT3--SPCA-1 siNC/siFUT3

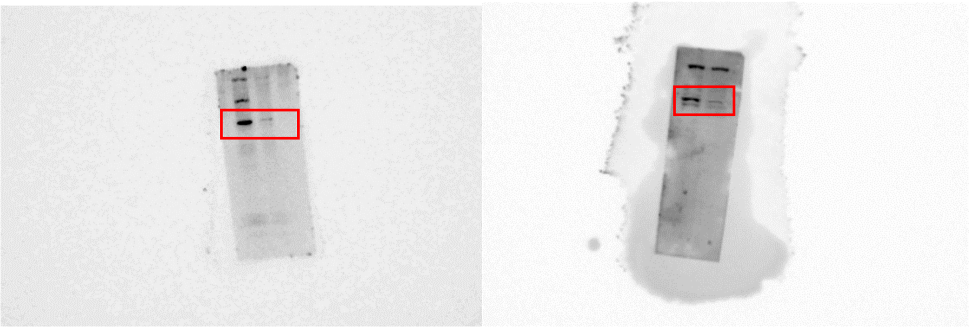

Figure 6c

GAPDH H1975 siNC/siFUT3--SPCA-1 siNC/siFUT3

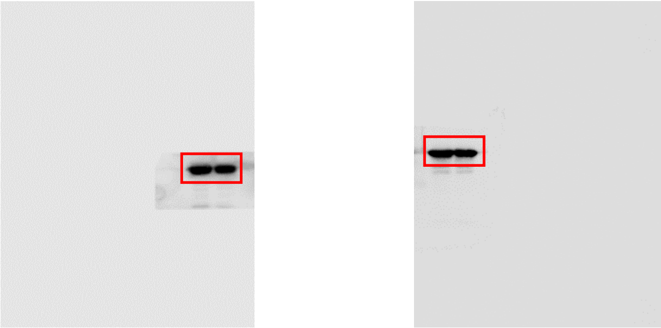

NF-κB H1975 siNC/siFUT3--SPCA-1 siNC/siFUT3

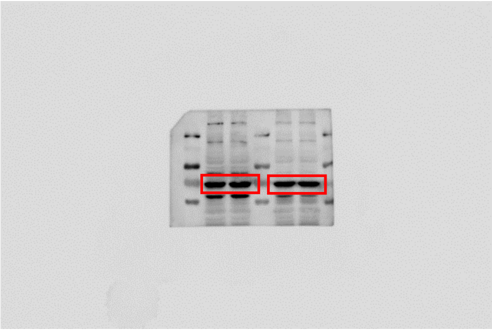

p-NF-κB H1975 siNC/siFUT3--SPCA-1 siNC/siFUT3

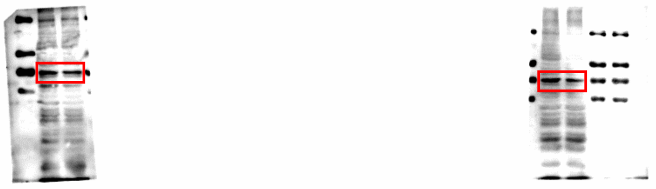

IκBα H1975 siNC/siFUT3--SPCA-1 siNC/siFUT3

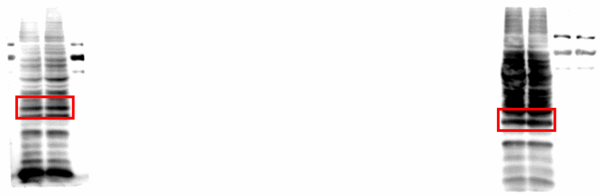

p-IκBα H1975 siNC/siFUT3--SPCA-1 siNC/siFUT3

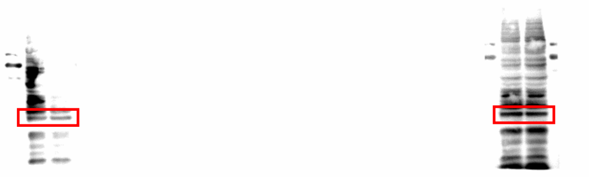

Supplement: Supplementary file 1 — Supplementary Material 1 [file 12890_2023_2688_MOESM1_ESM.pdf]
